# Supplementary material for: Influenza A Virus Infection Reactivates Human Endogenous Retroviruses Associated with Modulation of Antiviral Immunity
Source: Viruses. 2022 Jul 21;14(7):1591. doi: 10.3390/v14071591 (PMC9320126; doi:10.3390/v14071591)
Supplement: Supplementary file 1 [file viruses-14-01591-s001.zip › MotifAnalysis.zip!/MotifOutput_PR8M/homerResults/motif3.similar.html]

motif3

## Information for motif3

C
T
A
G
A
C
T
G
A
C
G
T
A
G
T
C
C
G
T
A
A
G
T
C
C
T
A
G
A
G
T
C
A
C
G
T
A
G
T
C
A
G
T
C
A
C
G
T
  
Reverse Opposite:  

C
G
T
A
A
C
T
G
A
C
T
G
C
G
T
A
A
C
T
G
A
G
T
C
A
C
T
G
A
C
G
T
A
C
T
G
C
G
T
A
G
T
A
C
A
G
T
C
  

|  |  |
| --- | --- |
| p-value: | 1e-21 |
| log p-value: | -4.871e+01 |
| Information Content per bp: | 1.928 |
| Number of Target Sequences with motif | 6.0 |
| Percentage of Target Sequences with motif | 26.09% |
| Number of Background Sequences with motif | 2.0 |
| Percentage of Background Sequences with motif | 0.00% |
| Average Position of motif in Targets | 161.8 +/- 14.7bp |
| Average Position of motif in Background | 135.8 +/- 51.8bp |
| Strand Bias (log2 ratio + to - strand density) | 10.0 |
| Multiplicity (# of sites on avg that occur together) | 1.00 |
| Motif File: | file (matrix) reverse opposite |

### Similar de novo motifs found

|  |  |  |  |  |  |  |  |
| --- | --- | --- | --- | --- | --- | --- | --- |
| Rank | Match Score | Redundant Motif | P-value | log P-value | % of Targets | % of Background | Motif file |
| 1 | 0.936 | A C G T A G T C C G T A A G T C A C T G A G T C A C G T A G T C A G T C A C G T | 1e-14 | -32.252519 | 21.74% | 0.02% | motif file (matrix) |
| 2 | 0.624 | A C T G C T G A A G T C C G T A G T A C A C G T A G T C A G C T | 1e-9 | -22.403994 | 65.22% | 9.87% | motif file (matrix) |
| 3 | 0.609 | A C T G G T A C A C G T A G T C A G T C A C G T A C T G C G T A | 1e-6 | -15.992388 | 30.43% | 1.79% | motif file (matrix) |
| 4 | 0.672 | A T C G A C G T T A C G G T C A A G C T A T C G A G T C G A C T | 1e-6 | -14.798876 | 21.74% | 0.66% | motif file (matrix) |
| 5 | 0.689 | A C G T A G T C G T A C A G T C A C T G A G C T C G A T A T G C | 1e-6 | -14.122533 | 30.43% | 2.37% | motif file (matrix) |
